# Conditional QTL mapping for waterlogging tolerance in two RILs populations of wheat

**DOI:** 10.1186/2193-1801-2-245

**Published:** 2013-05-27

**Authors:** Ma Yu, Guo-Yue Chen

**Affiliations:** Triticeae Research Institute, Sichuan Agricultural University, 211 Huimin Road, Wenjiang, Chengdu, Sichuan 611130 China

**Keywords:** QTL, Waterlogging tolerance, Conditional and unconditional mapping

## Abstract

Waterlogging is a widespread limiting factor for wheat production throughout the world, specially irrigated and high rainfall environments. Only few studies reported QTLs for waterlogging tolerance. To identify quantitative trait loci (QTLs) for waterlogging tolerance, root dry weight index (RDWI), shoot dry weight index (SDWI), total dry weight index (TDWI) were measured at seedling stage in two unrelated recombinant inbred lines (RILs) populations. These populations were International Triticeae Mapping Initiative (ITMI) population ‘W7984 / Opata85’, and ‘SHW-L1 × Chuanmai 32’ (SC) population. Conditional QTL mapping and unconditional QTL mapping were studied to dissect the genetic relationship between TDWI and its components of SDWI and TDWI. Total of 36 QTLs for waterlogging tolerance in ITMI population and 10 QTLs in SC population were identified in present study. Of them, 17 alleles from synthetic hexaploid wheat ‘W7984’ and 3 alleles from synthetic hexaploid wheat ‘SHW-L1’ contribute positively to waterlogging tolerance. Combinations of conditional and unconditional mapping methods indicate that SDWI showed tighter genetic correlation with TDWI than RDWI. This QTL identification study and dissection provide theoretical basis and application foundation to Marker-assisted selection (MAS) of waterlogging tolerance improvement in wheat.

## Background

Waterlogging is a widespread limiting factor for wheat production throughout the world specially irrigated and high rainfall environments. About 10–15 million ha of the world’s wheat growing areas are affected by waterlogging each year (Sayre et al. 1994), representing 15-20% of the 70 million ha annually cultivated for wheat production, especially in south and south-east Asia including Bangladesh, Pakistan, India, Nepal and China (Samad et al. 2001;Settler and Waters 2003). The breeding and cultivation of resistant wheat cultivars is the most promising strategy to reduce the risk of waterlogging. MAS could be a promising tool to facilitate the selection of resistant cultivars and to enhance breeding efficiency.

Waterlogging tolerance is defined as the survival or the maintenance of growth rates under waterlogging to nonwaterlogged conditions (Settler and Waters 2003). In previous studies, traits have been measured include plant height, growth increment, and shoot or root dry weight (Burgos et al. 2001;Boru et al. 2001;Qiu et al. 2007;Li et al. 2008;Parelle et al. 2010). In early stage of wheat, the maintenance of biomass in waterlogging is of major importance from agronomic view (Parelle et al. 2010). It also associated biomass distribution between overground and subterraneous organs. Therefore, maintenance of shoot dry weight, root dry weight, and total dry weight can be used as indicators for waterlogging tolerance in wheat.

Genetic diversity for waterlogging tolerance existed in wheat (Huang et al. 1994;Setter et al. 1999). However, QTLs for waterlogging tolerance have been reported in few studies (Poysa 1984;Taeb et al. 1993;Burgos et al. 2001;Boru et al. 2001). In addition, one dominant gene controlling tolerance of waterlogging was detected in both wheat and barley (Cao et al. 1995;Zhou et al. 2007). These studies indicate that waterlogging tolerance is a complex trait, and classical genetic studies are limited. More recently, a method for multivariable conditional analysis was proposed for analyzing the contributions of component traits to a complex trait and for investigating the genetic relationship between two traits at the QTL level (Wen and Zhu 2005). This method may dissect genetic relationships between maintenance ability of shoot dry weight (or root dry weight) and total dry weight under waterlogging condition.

In present study, we performed QTL detection for TDWI, RDWI, and SDWI at seedling stage in two unrelated recombinant inbred lines (RILs) populations, both of which were developed by hybrid with synthetic hexaploid wheat. Conditional study based on Wen and Zhu was also analyzed (Wen and Zhu 2005). The objectives of this study were to: (1) understand the genetic control of waterlogging tolerance. (2) specify the genetic relationships between maintenance ability of shoot dry weight (or root dry weight) and total dry weight under waterlogging at QTL level.

## Results

### Phenotypic summary

Significant dry weight loss in mean value was observed in ITMI and SC populations during waterlogging treatment. Commercial cultivar ‘opata85’ showed higher waterlogging tolerance index for SDW, RDW, and TDW than synthetic wheat ‘W7984’ in ITMI population (Table 1). However, synthetic wheat ‘SHW-L1’ showed higher SDWI and TDWI than Commercial cultivar ‘Chuanmai 32’ in SC population (Table 2). All investigated traits following waterlogging stress showed transgressive segregation in both RILs populations. All traits segregated continuously and followed a normal distribution in both populations. In correlation analysis, SDWI, RDWI, and TDWI showed significant positive correlation (Table 3).

**Table 1:** **Phenotypic variations of waterlogging tolerance index in ITMI population**

Trait^a^	Parents		RILs population						
	Opata85	W7984	Min.	Max.	Mean	SD	CV^b^	Skewness	Kurtosis
RDWI	0.18	0.07	0.07	0.30	0.18	0.21	0.30	0.44	1.20
SDWI	0.29	0.16	0.18	0.71	0.46	1.68	0.17	−0.25	−0.30
TDWI	0.47	0.23	0.25	0.97	0.64	0.95	0.25	−0.20	0.42

^a^ Traits were root dry weight index (RDWI), shoot dry weight index (SDWI), total dry weight index (TDWI).

^b^ Coefficient of variability.

**Table 2:** **Phenotypic variations of waterlogging tolerance index in SC population**

Trait^a^	Parents		RILs population						
	Chuanmai 32	SHW-L1	Min.	Max.	Mean	SD	CV^b^	Skewness	Kurtosis
RDWI	0.19	0.09	0.02	0.36	0.23	0.19	0.29	−0.35	−0.09
SDWI	0.32	0.49	0.19	0.81	0.45	0.17	0.25	−0.19	−0.42
TDWI	0.51	0.58	0.28	1.00	0.68	0.16	0.23	−0.16	−0.31

^a^ and ^b^ can referred to Table 1.

**Table 3:** **Correlation coefficient among waterlogging tolerance index and conditional traits in ITMI and SC populations**

	RDWI^a, b^	SDWI	TDWI	y_(TDWI|RDWI)_^c^	y_(TDWI|SDWI)_
RDWI	1.00	0.43**	0.73**	0.00	0.83**
SDWI	0.57**	1.00	0.91**	0.88**	0.00
TDWI	0.79**	0.96**	1.00	0.68**	0.41**
y_(TDWI|RDWI)_	0.00	0.82**	0.61**	1.00	−0.28**
y_(TDWI|SDWI)_	0.83**	0.02	0.31**	−0.56**	1.00

^a^ Data in lower left quarter of the matrix was the correlation coefficient in ITMI population whereas data in top right corner was the correlation coefficient in SC population.

^b^ ** significant r-values p < 0.01.

^c^ Conditional phenotypic values *y*_(TDWI|RDWI)_ or *y*_(TDWI|SDWI)_ indicate the value of TDWI without the influences of RDWI or SDWI, Other abbreviations for traits and environments can refer to Table 1.

### QTL mapping in ITMI population

**Figure 1:**
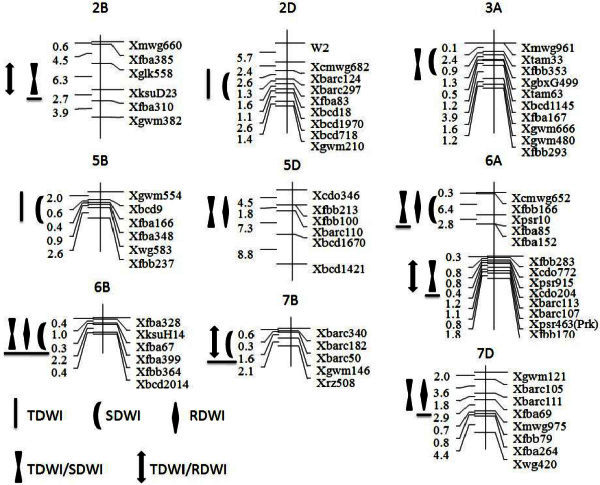
**Unconditional and conditional QTL mapping with significant LOD values in the ITMI population.** Only QTL clusters are shown in the figure. QTL with underline indicate that ‘W7984’ alleles reduce phenotypic value, whereas with no underline indicate ‘W7984’ alleles contributed positively. Abbreviations for traits and environments can refer to Tables 1 and 3.

Total of 36 QTLs were identified on 18 chromosomes in ITMI population, and they explained 0.8- 28.2% of the phenotypic variation (Table 4). Of these QTLs, 17 alleles from synthetic hexaploid wheat ‘W7984’ contributed positively to waterlogging tolerance, and the other 19 alleles from ‘Opata85’ contributed positively. Total of 10 common QTL regions were identified in present study, which carried 2 to 4 QTLs, especially the region of *XksuH14*-*Xfbb364* on 6B which carried QTLs for RDWI, SDWI, TDWI, and *y*_(TDWI|SDWI)_ (Figure 1).

**Table 4:** **Unconditional and conditional QTL mapping with significant LOD values in the ITMI population**

Chrom	Interval marker			R^2^/LOD^a^		
		RDWI	y_(TDWI|RDWI)_	SDWI	y_(TDWI|SDWI)_	TDWI
1A	*Xgwm357*-*Xbcd1407*			−12.3/7.3		
1B	*Xgwm550*-*Xgwm33*	−12.8/7.9				
1D	*Xpsr11*-*XksuD14*				−1.6/6.0	
	*Xgwm337*-*Xbarc229*	−6.6/4.6				
2A	*Xgwm47*-*Xgwm312*				−1.8/6.2	
2B	*Xgwm129*-*Xwg996*				4.9/15.4	
	*Xfba385*-*Xfba310*		28.2/10.2		−7.1/20.0	
2D	*Xcmwg682*-*Xbcd718*			5.4/2.5		6.9/2.6
3A	*Xtam33*-*Xgwm480*			12.9/5.4	7.9/19.8	
	*Xfbb322*-*XgbxG242*	18.0/10.3				
3B	*Xfba310*-*Xfbb293*	4.2/3.1				
3D	*Xfbb316*-*XksuE14*				1.2/4.6	
4B	*Xgwm538*-*Xfbb67*				−3.3/11.2	
4D	*Xfba177*-*Xgwm609*				−10.8/26.1	
5A	*Xbarc117*-*Xgwm129*				−7.1/19.7	
5B	*Xfba166*-*Xfba348*			9.8/4.4		9.2/3.4
5D	*Xfbb100*-*Xbcd1670*	13.7/8.6			19.2/28.2	
6A	*Xpsr10*-*Xfba85*	11.1/7.2		5.8/2.7	−3.5/11.1	
	*Xcdo772*-*Xfbb170*		8.4/3.8		−16.9/33.9	
6B	*XksuH14*-*Xfbb364*	−9.2/7.0		−6.5/3.2	−7.3/19.1	−11.2/4.0
6D	*Xbcd1319*-*XksuD1*				−2.2/8.1	
7B	*Xbarc50*-*Xgwm146*		−17.0/7.5	−10.8/4.8		
7D	*Xbarc105*-*Xwg420*	−6.7/4.4			0.8/3.1	

^a^ R^2^ is the percentage of phenotypic variance. LOD is the LOD peak of the QTL. Negative signs indicate that ‘W7984’ alleles reduce phenotypic value whereas positive indicate that ‘W7984’ alleles increase the phenotypic value. Abbreviations for traits can refer to Tables 1 and 3.

Total of 18 QTLs were detected on 12 chromosomes in unconditional analysis, 8 of which were identified for RDWI, 7 QTLs were found for SDWI, and the rest 3 QTLs were detected for TDWI (Table 4). For these three investigated traits, 4 alleles from ‘Opata85’ contributed positively to RDWI, 3 alleles from ‘W7984’ contributed positively to SDWI, and 2 alleles from ‘W7984’ contributed positively to TDWI. Phenotypic variances explained by these QTLs varied from 4.2- 18.0%.

For conditional analysis, 18 QTLs were identified on 13 chromosomes (Table 4). Of these QTLs, 3 QTLs were identified for *y*_(TDWI|RDWI)_, and the rest 15 QTLs were identified for *y*_(TDWI|SDWI)_. For these two conditional traits, 2 alleles from ‘W7984’ contributed positively to *y*_(TDWI|RDWI)_, and 10 alleles from ‘Opata85’ contributed positively to *y*_(TDWI|RDWI)._ Phenotypic variances explained by these QTLs varied from 0.8- 28.2%.

### QTL mapping in SC population

**Figure 2:**
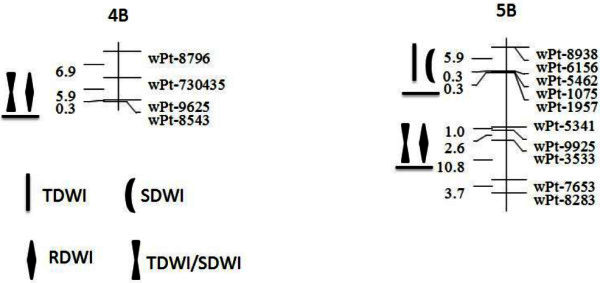
**Unconditional and conditional QTL mapping with significant LOD values in the SC population.** Only QTL clusters are shown in the figure. QTL with underline indicate that ‘SHW-L1’ alleles reduce phenotypic value. Abbreviations for traits and environments can refer to Tables 1 and 3.

Total of 10 QTLs were identified on 6 chromosomes in SC population, and they explained 5.3- 29.3% (Table 5). Of these QTLs, 3 alleles from synthetic hexaploid wheat ‘SHW-L1’ contributed positively to waterlogging tolerance, and the other 7 alleles from ‘Chuanmai 32’ contributed positively. Total of 3 common QTL regions were identified in present study (Figure 2). These regions were located on 4B and 7B, and every region carried two QTLs.

**Table 5:** **Unconditional and conditional QTL mapping with significant LOD values in the SC population**

Chrom	Interval marker			R^2^/LOD^a^		
		RDWI	y_(TDWI|RDWI)_	SDWI	y_(TDWI|SDWI)_	TDWI
3D	*wPt*-*671868*-*wPt*-*741417*				29.3/23.0	
4B	*wPt*-*730435*-*wPt*-*9625*	−7.8/3.			−5.3/3.0	
5B	*wPt*-*6014*-*wPt*-*2607*	−11.7/2.6				
6B	*wPt*-*5037*-*wPt*-*9231*					6.0/2.7
6D	*wPt*-*730822*-*wPt*-*733873*					11.2/3.0
7B	*wPt*-*6156*-*wPt*-*5462*			−5.7/2.5		−5.9/2.7
7B	*wPt*-*3533*-*wPt*-*7653*	−13.2/4.7			−6.4/3.3	

^a^ Negative signs indicate that ‘SHW-L1’ alleles reduce phenotypic value whereas positive indicate that ‘SHW-L1’ alleles increase the phenotypic value. Abbreviations for traits and environments can refer to Tables 1 and 3. Title description can refer to Table 4.

For unconditional analysis, 7 QTLs were identified 5 chromosomes (Table 5). Of these QTLs, 3 of which were identified for RDWI, only one QTL was detected for SDWI, and the rest 3 QTLs were found for TDWI. For these three investigated traits, all alleles from ‘Chuanmai 32’ contributed positively to phenotypic variation, except QTLs for TDWI on 6B and 6D. Phenotypic variances explained by these QTLs varied from 5.9- 13.2%.

For conditional analysis, none QTLs were detected for *y*_(TDWI|RDWI)_, and 3 QTLs were identified for *y*_(TDWI|SDWI)_ (Table 5). These QTLs were located on 3D, 4B, and 7B, with phenotypic explanation of 29.3%, 5.3% and 6.4%. For QTLs on 3D, alleles from ‘SHW-L1’ contributed positively to phenotypic variation. Whereas the rest alleles from ‘Chuanmai 32’ contributed positively to *y*_(TDWI._

### Genetic relation between RDWI (or SDWI) and TDWI

Biologically, the value of TDWI equals RDWI plus TDWI. There were 5 results when performing conditional QTL mapping analysis, such as the conditional QTL analysis of TDWI conditioned on RDWI: 1) a QTL detected for TDWI can be also identified for RDWI with a similar or equal effect, but can’t identified effect for *y*_(TDWI|RDWI)_, this indicate that this QTL for TDWI is entirely contributed by RDWI. 2) a QTL for TDWI was identified for RDWI and *y*_(TDWI|RDWI)_, this suggest that this QTL for TDWI is partly contributed by RDWI. 3) a QTL for TDWI only identified for *y*_(TDWI|RDWI)_, this indicate that this QTL for TDWI is independent of RDWI. 4) an additional QTL only identified for *y*_(TDWI|RDWI)_, which means that the expression of the QTL for TDWI is contributed by SDWI or suppressed by RDWI. 5) a QTL only identified for RDWI, this means that this QTL for RDWI have no effect on TDWI.

Of the 3 QTLs for TDWI in ITMI population, 2 of which were entirely contributed by SDWI and the rest QTL was partly contributed by SDWI and RDWI. In SC population, 2 QTLs for TDWI were independent to SDWI and RDWI, and 1 QTL was entirely contributed by SDWI.

Total of 8 QTLs for RDWI in ITMI population were identified, only 1 of which entirely contributed to expression of the QTL for TDWI, 3 QTLs showed genetic coordination with SDWI to suppressed the expression of QTL for TDWI, and the rest 4 QTLs were independent to TDWI. In SC population, 3 QTLs for RDWI were identified, 2 of which coordinated with SDWI to suppressed the expression of QTL for TDWI.

For SDWI, 7 QTLs were identified in ITMI population, 3 of which showed contribution to TDW, 2 QTLs were suppressed the expression of QTL for TDWI, and the rest 2 QTLs have no effect on TDWI. Only one QTL was detected for SDWI in SC population, and this QTL affected TDWI independently.

## Discussion

### QTL mapping

Although no significant difference for RDWI existed among two commercial parents and two synthetic parents between two RILs populations, QTLs number for RDWI in ITMI population was more than that in SC population. QTLs number variation also existed for SDWI, *y*_(TDWI|SDWI)_, and *y*_(TDWI|SDWI)_ in ITMI population and SC population. For identified QTLs, chromosome locations were also different in two populations, except QTLs for *y*_(TDWI|SDWI)_ on 3D and 4B, and QTL for total dry weight index on 6B (Table 4, Table 5). These identification variations might due to the different genetic background among two populations.

To our knowledge, there were only few studies reported QTLs for waterlogging tolerance (Taeb et al. Taeb et al. 1993;Poysa 1984;Burgos et al. 2001). Boru et al. found that up to 4 genes controlled waterlogging tolerance (Boru et al. 2001). However, this study lacks genetic map as framework to identify chromosome location. In previous studies, QTLs for waterlogging were reported on homologous group 3, 4 and 5 (Taeb et al. 1993;Poysa 1984;Burgos et al. 2001). Ma et al. identified a QTL associated with salt tolerance through measurement of shoot weight index (Ma et al. 2007). We also identified same chromosome locations in ITMI population and SC population, except 4A (Table 4, Table 5).

Genetic relation analysis between RDWI, SDWI and TDWI indicated that SDWI showed tighter genetic correlation with TDWI than RDWI in both ITMI population and SC population. Several QTLs for RDWI were coordinated with SDWI to affect the expression of QTL for TDWI, and most of them showed suppression. Breakthrough these suppressed relationship will be notable improvement for waterlogging breeding in wheat.

### Utilization of synthetic wheat in wheat improvement for waterlogging tolerance

Common wheat is considered as one of the most intolerant crop for waterlogging (Thomson et al. 1992). Utilization of wide hybridization with wild relatives in Triticeae to improve waterlogging tolerance should be a feasible way (King et al. 1997;Colmer et al. 2006;Munns et al. 2011). Although synthetic wheat ‘W7984’ showed poor phenotype in waterlogging treatment, we still identified 17 alleles from synthetic hexaploid wheat ‘W7984’ contributed positively to waterlogging tolerance. In addition, 3 alleles from synthetic hexaploid wheat ‘SHW-L1’ also were found contributed positively to waterlogging tolerance. These QTLs might be novel resources which can improve waterlogging tolerance for common wheat.

## Conclusions

This study identified 36 QTLs for waterlogging tolerance in ITMI population, and 10 QTLs in SC population. Combinations of conditional and unconditional mapping methods dissect the genetic relationship between QTL for TDWI and its components. This QTL identification study and dissection provide theoretical basis and application foundation to MAS of waterlogging tolerance improvement in wheat.

## Methods

### Populations used for QTL analysis

The first population is the International Triticea Mapping Initiative (ITMI) population ‘W7984 / Opata’, which consists of 112 recombinant inbred lines (RILs) (Van Deynze et al. 1995). The female parent ‘W7984’ is synthetic hexaploid wheat and the male parent ‘Opata85’ is a commercial cultivar. The second population (SC) of 171 recombinant inbred lines derived from the cross between ‘SHW-L1’ and ‘Chuanmai 32’. The parents, Synthetic hexaploid wheat ‘SHW-L1’, was obtained through distant hybridization between accession AS2255 (AABB, *T*. *turgidum* ssp. *turgidum*) and AS60 (DD, *Ae*. *tauschii* ssp. *tauschii*) (Zhang et al. 2004), and ‘Chuanmai 32’ was a commercial wheat cultivar at southwest of China. Both parents of the synthetic wheat are sensitive to waterlogging, whereas both parents of the common wheat are tolerant to waterlogging in those two populations. All the materials were provided by Triticeae Institution, Sichuan Agricultural University, China.

### Evaluation of waterlogging tolerance

Two replicates each with 20 disinfectant seeds which had been pre-selected for uniform mass. Seeds were germinated in water into plastic pots (78.5 cm square by 15.8 cm in height) containing quartz gravel. After germination, 10 plants were kept in each pot and grown in a glasshouse under 16 hour’s illumination a day. All pots for each replicate were placed in a single large plastic pool. Temperature was controlled at 18°C to 20°C. Waterlogging was achieved by filling the pool with water to make sure that the tallest leaf tip was at least 10 cm below the water surface. Watelogging treatment was conducted after 7 days of germination, and continued for 7 days. After that, water level was treated the same as control in the experiments. Following the treatment, all plants in both control and treatment were cultured with modified Hoagland’s nutrient solution in one week. Root dry weight, shoot dry weight, and total dry weight were measured. Waterlogging index calculated as values in waterlogging treatment divided by total dry weight in control.

### Statistical analysis

Variance and covariance components were first analysised based on MINQUE method proposed by Zhu (1992). To estimate genotypic effect of the three traits via QGAStation 1.0 (http://ibi.zju.edu.cn/software/qga/), data were assembled according to format of QTLData. QTLData menus of QGAStation 1.0 were selected followed Cui et al. (2013). Conditional phenotypic values *y*_(TDW|SDW)_ or *y*_(TDW|RDW)_ indicate the value of TDWI without the influences of SDWI or RDWI.

### Genetic map and QTL analysis

Map date was downloaded from http://wheat.pw.usda.gov for ITMI population (Song et al. 2005). This map contained 1,410 loci (SSR and AFLP), covered 2, 541 cM of genetic distance, with marker density of 1.72 cM/marker in total.

For ‘SHW-L1 × Chuanmai 32’ (SC) population, two marker systems, DArT and SSR, were used for linkage map construction. A wheat DArT array consisting of 7,000 random markers were used for genotyping of the parents. DArT array was carried out by the Triticarte Pty. Ltd. (http://www.triticarte.com.au/). Procedures of hybridization of genomic DNA to the DArT array, image analysis and polymorphism scoring were as described by Akbari et al. (2006). Sixty-eight SSR markers polymorphic between two parents of the mapping population were also used for the linkage map construction. The genetic linkage map was constructed with JoinMap (version 4.0) (Van Ooijen, 2006), thresholds of recombination frequency from 0.05 to 0.20 were tested, and until a threshold with the optimum number of markers in linkage groups maintaining linkage order and distance was obtained. Known chromosomal locations of the SSR and DArT markers were used to assign linkage groups to specific chromosomes. ML (Maximum Likelihood) mapping function was used to order the loci belong one chromosome.

Inclusive composite interval mapping by IciMapping 2.0 was used based on stepwise regression of simultaneous consideration of all marker information (Li et al. 2007). The walking speed for all QTLs was 1.0 cM, and LOD threshold set as 2.5. Both the observed phenotypic valuesand the conditional phenotypic values were used in QTL mapping analyses.

## Abbreviations

QTLs: Quatitative trait loci
RDWI: Root dry weight index
SDWI: Shoot dry weight index
TDWI: Total dry weight index
RILs: Recombinant inbred line
ITMI: International triticeae mapping initiative population ‘W7984 / Opata85’
SC: ‘SHW-L1 × Chuanmai 32’ population
MAS: Marker-assisted selection
